# The size of human subcutaneous adipocytes, but not adiposity, is associated with inflammation, endoplasmic reticulum stress, and insulin resistance markers

**DOI:** 10.1007/s11033-023-08460-y

**Published:** 2023-05-23

**Authors:** Sara Pourdashti, Nassim Faridi, Forouzandeh Monem-Homaie, S. Hamid Yaghooti, Ahmadreza Soroush, S. Zahra Bathaie

**Affiliations:** 1 Department of Clinical Biochemistry, Faculty of Medical Sciences, Tarbiat Modares University (TMU), P.O. Box: 14155-331, Tehran, Iran; 2 Obesity and Eating Habits Research Center, Endocrinology and Metabolism Molecular- Cellular Sciences Institute, Tehran University of Medical Sciences, Tehran, Iran; 3 UCLA-DOE Institute, University of California, Los Angeles (UCLA), CA USA

**Keywords:** Obesity, Adipogenesis, Angiogenesis, ER stress, sXBP-1, PPARγ*2*, WNT10B, HOMA-IR

## Abstract

**Background:**

The fat storage capacity of the adipose tissue prevents ectopic lipid deposition, which is one of the risk factors for metabolic abnormalities in obesity. This capacity depends upon the adipogenic gene expression and blood supply provision for tissue expansion through angiogenesis. Here, we studied hyperplasia/hypertrophy of subcutaneous white adipose tissue (scWAT) concerning adipogenic gene expression, angiogenic status, and metabolic parameters in non-obese and different classes of obese individuals.

**Methods:**

The scWAT samples were collected from 80 individuals. The anthropometric parameters, adipose tissue cell size, serum biochemistry, ER stress-induced XBP1 splicing, PPARγ2, SFRP1, WNT10B, and VEGFA gene expression levels were studied. In addition, the CD31 level was investigated by Western blotting.

**Results:**

The obese individuals had greater waist circumferences and higher serum TG, TC, insulin, and HOMA-IR than the non-obese group. However, the largest adipocyte size, increased TNFα, insulin, and HOMA-IR, and the highest expression level of *sXBP1*, *WNT10B*, and *VEGFA* were observed in Class I obese individuals. It means that inflammation, insulin resistance, and ER stress accompany hypertrophic scWAT adipocytes with limited adipose tissue expansion ability. Furthermore, the Class II + III obese individuals showed high *PPARγ2* expression and CD31 levels. There is adipogenesis through hyperplasia in this group. The *SFRP1* expression was not significantly different in the studied groups.

**Conclusion:**

The results suggest that the capability of adipogenesis with inadequate angiogenesis is related to the metabolic status, inflammation, and ER function. Therefore, therapeutic strategies that support both angiogenesis and adipogenesis can effectively prevent the complications of obesity.

## Introduction

Obesity is a common global health problem. The excessive fat deposition can be in subcutaneous white adipose tissue (scWAT) or visceral adipose tissue. Adipose tissue is a primary metabolic organ essential for energy homeostasis and insulin sensitivity [1]. The lipid storage capacity of expanding adipose tissue prevents lipid deposition in tissues such as muscle and liver in case of inadequate lipid metabolism. It prevents fat mounting in these tissues and the resultant inflammation, insulin resistance, and metabolic dysfunction [2, 3]. Uncontrolled fat deposition in adipose tissue is usually associated with metabolic disorders such as insulin resistance and dyslipidemia. Therefore, unraveling the subcutaneous adipose tissue development mechanisms could identify adipose tissue targets for treating metabolic diseases.

Angiogenesis is a major molecular event pivotal in adipose tissue enlargement. It is mainly regulated by the vascular endothelial growth factors (VEGFs) secretion and stimulation of endothelial cell proliferation of the existing blood vessels [4, 5]. Due to the excess caloric intake, adipose tissue can undergo rapid expansion. Due to the inability of the vasculature to keep pace with tissue growth, like a rapidly expanding tumor mass, obese adipose tissue becomes hypoxic. The hypoxic condition causes an increase in the level of hypoxia-inducible factor-1α (HIF-1α) expression. It has been reported that HIF1α induces fibrosis and inflammation in adipose tissue. [6]. Therefore, alternative mechanisms, other than HIF-1α, are involved in the stimulation of VEGFA production in adipose tissue.

Recent studies have shown the role of unfolded protein responses (UPR) due to the endoplasmic reticulum (ER) stress and Wnt signaling pathways in the process of angiogenesis in different tissues [7, 8]. The X-box 1 binding protein (XBP1) is a central component of the UPR pathway following ER stress, activating target gene expression as a transcription factor. In this process, XBP1 mRNA splicing to the active sXBP1 form mediates the production of proteins associated with protein folding and endoplasmic reticulum biogenesis [9]. In addition, XBP1 is involved in many biological processes beyond the unfolded protein responses, such as cellular differentiation, metabolism, insulin activity, and inflammation [10]. The sXBP1 was also associated with angiogenesis by binding to the VEGFA promoter and transcription initiation in different tissues. However, the angiogenic effect of this protein in adipose tissue is unknown [8].

Among the numerous Wnt pathway ligands in adipose tissue, WNT10b is the dominant ligand. The binding of a WNT-protein ligand to a Frizzled family receptor activates Wnt pathways that lead to the regulation of gene transcription through β-catenin activation. The Wnt pathways govern many biological processes [11]. WNT10b regulates adipogenesis in adipose tissue by inhibiting adipogenic transcription factors associated with obesity [12, 13]. Besides, the Wnt pathway is active in endothelial cells, and β-catenin binding sites in the VEGFA gene promoter regulate endothelial cell proliferation and angiogenesis [14]. Wnt signaling is antagonized at several levels. One group of these antagonists secreted frizzled-related proteins (SFRPs). At least five structurally similar SFRPs have been identified. One is SFRP1, whose expression is altered in obese murine and human scWAT. The SFRP1_/_ mice display reduced fat mass [15].

This study aimed to investigate adipose tissue expansion (adipogenesis level) and adipocytes size concerning *PPARγ2 and WNT10b* gene expression as regulators of adipogenesis; XBP1 expression as a marker of ER stress; angiogenesis parameters in the scWAT; and metabolic status of individuals with different levels of body mass index (BMI). Herein, we examined if different adipogenesis vs. adipocyte hypertrophy capacities in different individuals can determine the ER stress, angiogenesis, and metabolic status.

## Materials and methods

### Individuals

Eighty individuals aged 19 to 62 were selected from the patients undergoing elective abdominal surgery at the Shariati Hospital in Tehran, Iran. The participants had no underlying diseases. Participants’ height, weight, BMI, and waist circumference (WC) were determined before the surgery. In addition, subcutaneous adipose tissue samples were collected during the surgical procedure at the incision site. Written informed consent was obtained from all patients to be included in the study. The Tarbiat Modares University Ethics Committee approved the study with the following reference: IR.TMU.REC.1395. 491.

### Measurement of subcutaneous abdominal adipocyte size

Collected scWAT samples were fixed in formalin 4% for 24 h. Then, the tissues were embedded in paraffin, and sections of 3 μm were prepared and transferred onto slides for Hematoxylin and Eosin (H&E) staining. Digital images of the stained slides were prepared with 200x magnification using the Leica Qprodit imaging software on a light microscope (Leica Microsystems, Vlierweg, Netherlands). To obtain the adipose tissue cell size, 150 adipocytes over 15 different zones on a slide were randomly selected, and their size was measured using ImageJ software (v1.51a). The mean sizes of the measured cells were reported as the subcutaneous abdominal adipocyte size.

### Biochemical and inflammatory markers

The venous blood sample was obtained on the day of the biopsy after an overnight fast of at least 10 h. Biochemical measurements include fasting blood glucose (FBG), total cholesterol (TC), triglycerides (TG), high-density lipoprotein cholesterol (HDL-C), and low-density lipoprotein cholesterol (LDL-C) were determined with an autoanalyzer (Hitachi 917, Boehringer Mannheim, Marburg, Germany) using colorimetric assay methods. In addition, serum insulin (Cat. No: 2425-300 A, AccuBind, Netherlands), TNFα (ab46087, Abcam), and IL10 (ab46034, Abcam) were measured by Human ELISA kits. The homeostasis model assessment of insulin resistance (HOMA-IR) index was calculated using the following formula: Fasting insulin (in mU/ml) × Fasting glucose (in mmol/l) /22.5.

### Real-time PCR (qPCR)

A 300 mg frozen subcutaneous adipose tissue was homogenized with a Sonicator (S3000–010; Misonix, Inc., Farmingdale, NY, USA). Total RNA was extracted from adipose tissue and adipocytes using the RNeasy Lipid Tissue Mini Kit (Cat. No: 74,804, QIAGEN Science, Germany). RNA concentration was determined based on the absorbance at 260 nm on a Nano Photometer (Thermo Scientific, Wilmington, DE). For this purpose, 500 ng of the total RNA was reverse transcribed to cDNA using PrimeScriptTM RT reagent Kit (Cat. No: RR037A, Takara, Japan) containing oligo dT and random hexamer primers. Then, 2 ng of cDNA per gene was amplified using the SYBR® Premix Ex Taq ™ master mix (Cat. No. RR041A, Takara, Japan). The conditions for cDNA synthesis were one cycle of 37 °C for 15 min, one cycle of 85 °C for 5 s, and target sequence amplification conditions composed of an initial denaturation step at 95 °C for 30 s followed by 40 cycles of 95 °C for 5 s, 60 °C for 30 s. Real-time quantitative PCR was performed with the Applied Biosystem Thermocycler (StepOnePlus™, ABI, USA). Table 1 shows the primers used in this study.

**Table 1:** The forward and reverse primers were used in this study

Gene	Primers	
	Forward	Reverse
*PPARγ2*	5′-GGGTGAAACTCTGGGAGATTCTC-3′	5′-GATGCCATTCTGGCCCAC-3′
*SFRP1*	AGATGCTTAAGTGTGACAAGTTCC	TCAGATTTCAACTCGTTGTCACAG
*WNT10B*	5′- AGAGCCCCTTTAACCCTGATTC-3′	5′-ACTGTCTCCCATTATCCCAC-3′
*VEGFA*	5′TCACAGGTACAGGGATGAGGACAC-3′	5′-AAAGCACAGCAATGTCCTGAAG-3′
*18s RNA*	5′-GGCCCTGTAATTGGAATGAGT-3′	5′-CAAGATCCAACTACGAGCTT-3′

### Reverse transcription-PCR analysis

Reverse Transcriptase-PCR analysis of total RNA was performed using MJ mini thermal cycler (PTC-1148 Bio-Rad, Hercules, CA, USA) to assess the XBP1 splicing and simultaneous detection of both sXBP1 and uXBP1 mRNA, using the following primers. Forward primer: TTACGAGAGAAA AC TCATGGCC, Reverse primer: GGGTC CAAGTTGTCCAGAATGC. PCR product sizes were as follows: uXBP1 = 289 bp, sXBP1 = 263 bp. The XBP1 mRNA splicing was evaluated using RT-PCR. For this purpose, cDNA was subjected to PCR amplification using Taq Polymerase (30 cycles, denaturation 1 min at 94 °C, hybridization 1 min at 60 °C, and elongation 1 min at 72 °C). This situation also detected hybrid XBP1 (hXBP1) combined from two forms of XBP1, sXBP1 and uXBP1. PCR products were then resolved by 2% agarose gel electrophoresis and visualized using ethidium bromide and UV transillumination. The percent of sXBP1 was calculated by Eq. 1 [16].

Eq. 1 $$\text{s}\text{X}\text{B}\text{P}1\text{\%}= \frac{\text{s}\text{X}\text{B}\text{P}1 + 0.5 \text{h}\text{X}\text{B}\text{P}1}{\text{s}\text{X}\text{B}\text{P}1 + \text{h}\text{X}\text{B}\text{P}1+\text{u}\text{X}\text{B}\text{P}1}\times 100$$

### Western blotting

For Western blot analysis, total protein extraction from scWAT biopsies was performed using an ice-cold radioimmunoprecipitation assay (RIPA) buffer containing protease inhibitors. First, the total protein content was measured using the Bradford protein assay. Thus, 30 µg of extracted proteins were loaded on 10% SDS–PAGE and transferred to PVDF membranes (3,010,040,001, Amersham, USA) by electroblotting using the BioRad transfer system. The membranes were blocked with 5% nonfat dry milk in PBST (phosphate-buffered saline containing 0.01% Tween-20) for 1 h at room temperature. Next, they were incubated in a 1:500 and 1:100 dilution of the primary mouse anti-human CD31 (Santa Cruz, USA) 4 and primary rabbit antibody anti-human β-actin (ab227387, Abcam Inc., Cambridge, MA) antibodies, respectively, overnight at 4 ºC. Next, the membranes were washed with PBST three times and incubated with a 1:2000 dilution of horseradish peroxidase-conjugated secondary goat anti-mouse IgG (170–6516, Bio-Rad). Finally, the Western blot bands were pictured following an enhanced chemiluminescence detection system (11,500,708,007, Roche, USA) on radiography films. Then, the photos were scanned and analyzed using Image J software (v.1.41).

### Statistical analysis

Data are provided as means ± SD. The SPSS version 19.0 statistical software was used for statistical analysis. The skewed distribution in some variables was transformed logarithmically. The independent t-test and one-way ANOVA tests were used to compare the means. In addition, Pearson’s correlation analysis was used to assess associations between variables. To compare the parameters of the individuals based on BMI, they were first divided into two non-obese (BMI < 30) and obese (BMI ≥ 30) groups [17]. We further divided the obese group based on BMI into three groups. So, individuals with BMI < 35 as Class I obese, individuals with 35 ≤ BMI < 40 as Class II, and individuals with (BMI≥40) as Class III. However, because significant differences between some parameters in Class I (mild obese individuals) with two other obese classes has been known as severely obese individuals [18, 19], we named the latter as Class II + III. P-value ≤ 0.05 was considered statistically significant.

## Results

### Anthropometric and biochemical parameters

Based on data in Table 1 S, the obese individuals had greater BMI (36.32 ± 5.72, 24.73 ± 2.77 P = 0.0001) and WC (87.78 ± 8.84, 102.58 ± 10.58 P = 0.0001) than the non-obese group. They also had higher serum TG (2.04 ± 0.16, 2.17 ± 0.21 P = 0.01), TC (2.19 ± 0.08, 2.25 ± 0.11 P = 0.04), insulin (1.21 ± 0.24, 1.36 ± 0.22 P = 0.01) and HOMA-IR (0.62 ± 0.28, 0.81 ± 0.22 P = 0.01) levels than the non-obese group.

Table 2 shows that insulin and HOMA-IR were only significantly higher in Class I obese individuals than in non-obese individuals (p = 0.03). However, no significant differences were observed between Class II and III, which we named Class II + III. On the other hand, among the metabolic parameters, TC levels were significantly higher in Class II + III obese groups than in non-obese (p = 0.01) group. There were no significant differences between non-obese and Class I obese groups in TC.

**Table 2:** The clinical characteristics of the individuals according to the obesity classes

	Non-Obese	Class I obesity	Class II + III obesity
Age (years)	45.50 ± 14.73	45.41 ± 16.47	47.2 ± 10.17
Gender (male/female)	23/21	7/13	8/10
WC (cm)	87.78 ± 8.84	98.94 ± 8.4 α	109.4 ± 11 β
WHR	0.87 ± 0.06	0.88 ± 0.05	0.87 ± 0.06
TG (mg/dl)ª	2.04 ± 0.16	2.15 ± 0.25	2.20 ± 0.17
TC (mg/dl)ª	2.19 ± 0.08	2.20 ± 0.08	2.29 ± 0.15 γ
HDL-C (mg/dl)	38.68 ± 10.69	35.20 ± 8.3	40.54 ± 7.43
LDL-C (mg/dl)ª	1.93 ± 0.16	1.96 ± 0.18	1.98 ± 0.21
Insulin (µU/ml)ª	1.21 ± 0.24	1.40 ± 0.23 δ	1.29 ± 0.19
HOMAª	0.62 ± 0.28	0.81 ± 0.24 η	0.79 ± 0.21

All data are presented as the means ± SD.

ª: Data are transformed logarithmically

α: Class I obese compred to non-obese p = 0.0001

β: Class II + III obese compred to non-obese p = 0.0001

γ: Class II + III obese compred to non-obese p = 0.01

δ, η: Class I obese compred to non-obese p = 0.03

### The Class I obese showed the largest adipocyte size

**Fig. 1:**
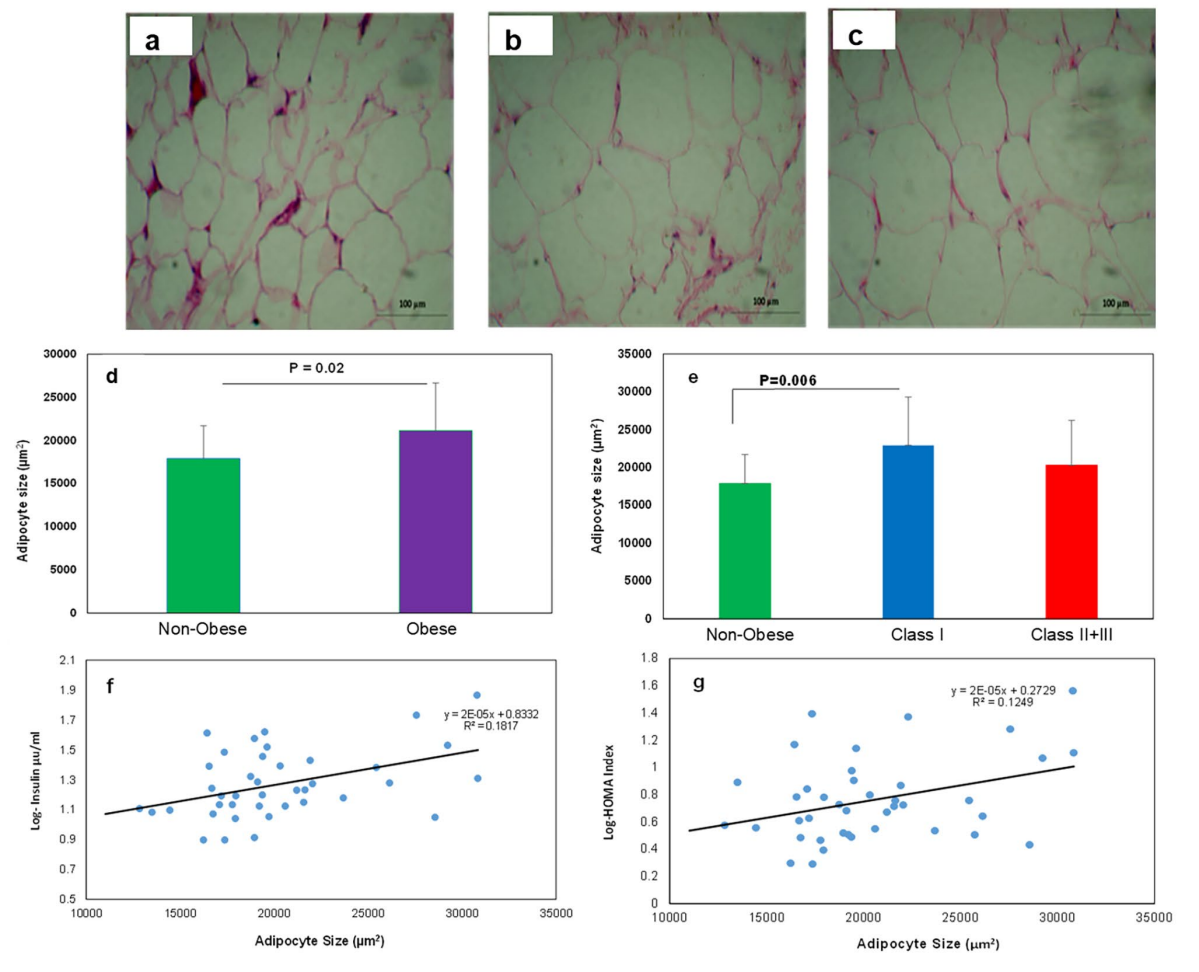
**Subcutaneous white adipose tissue of obese and non-obese individuals**. Representative hematoxylin and eosin images of scWAT sections of (**a**) non-obese, (**b**) Class I Obese, and (**c**) Class II + III Obese groups, magnification ⋅200. (**d** and **e**) Quantification of scWAT adipocyte size in different groups. (**f** and **g**) Correlation between serum Insulin and HOMA-IR with the adipocytes size, respectively. All data are shown as mean ± SEM. The level of significance is shown in the histograms

Figure 1a and b, and 1c show the subcutaneous abdominal adipocytes of the individuals. Figure 1d indicates that the adipocyte size of the obese individuals (all three Classes) was significantly larger than that of the non-obese individuals (21,144 ± 5526 vs. 17,892 ± 3800 µm^2^, p = 0.02). When the obese group was divided into subclasses (Fig. 1e), we observed that only adipocytes in the Class I obesity were significantly larger than the non-obese group (p = 0.006). However, the difference in cell size was not significant between the non-obese group and the severely obese group (Class II + III).

In addition, Fig. 1f g show the association between adipocyte size with serum insulin (R = 0.42 p = 0.006), and HOMA index (R = 0.35 p = 0.025), respectively.

### The expression of adipogenic-related genes

**Fig. 2:**
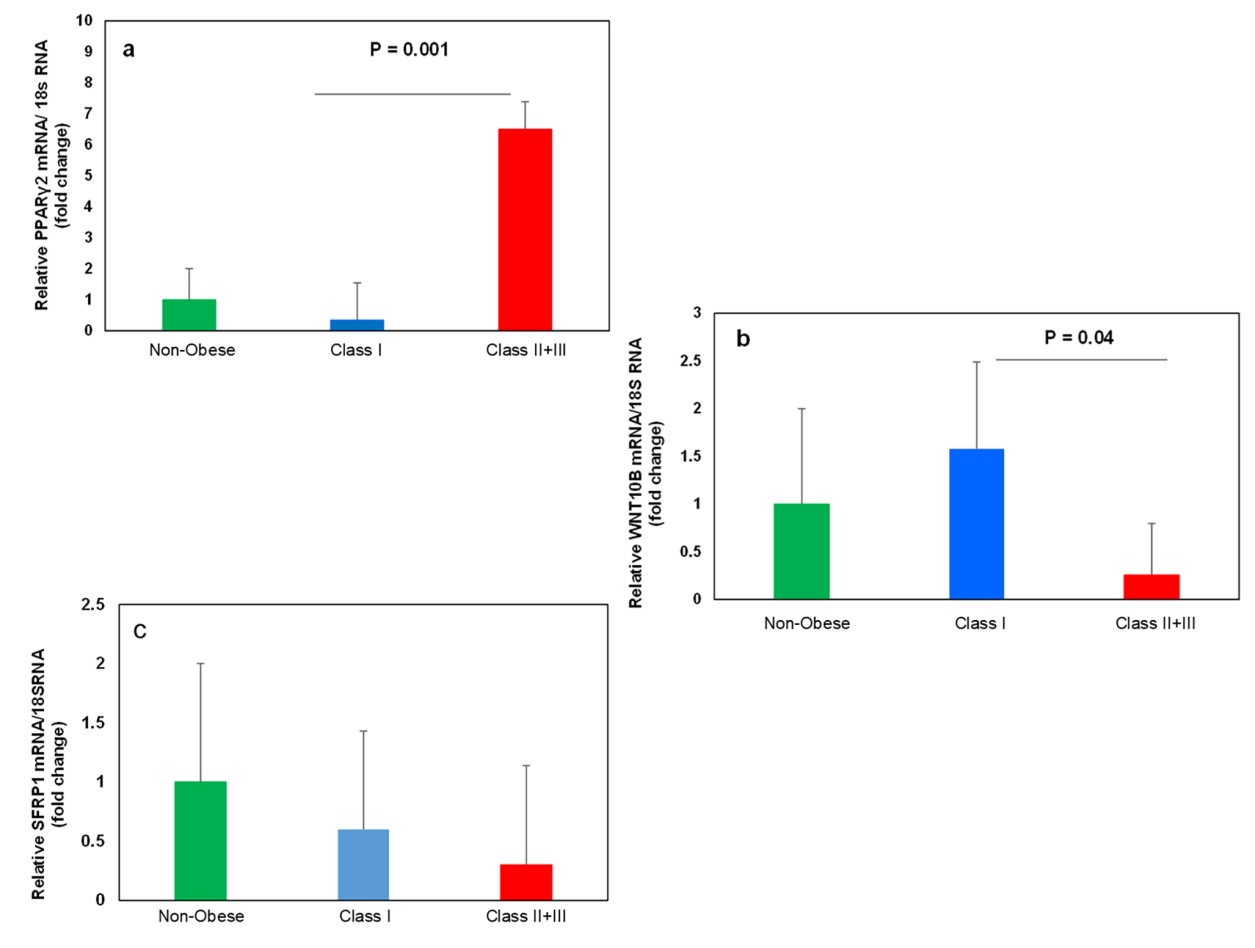
**PPARγ2, Wnt10b, and SFRP1 gene expression in scWAT.** The expression of these genes was measured by quantitative PCR and normalized to 18 s RNA. (**a**, **b**, and **c**) Relative mRNA expression of *PPARγ2*, *WNT10B*, and *SFRP1*in scWAT of Obese and non-obese individuals, respectively. All data are shown as mean ± SEM. The level of significance is shown in the histograms

The *PPARγ2 and SFRP1* expressions as two positives and *WNT10B* as a negative regulator gene of adipogenesis were evaluated in the scWAT of the individuals with different classes of obesity. Figure 2a shows the *PPARγ2* expression was significantly higher in the Class II + III obesity group than in other groups (p = 0.001). However, the expression level of *PPARγ2* was the same in the non-obese and Class I obesity groups. Figure 2b also shows a significantly higher expression of *WNT10B* in the Class I obesity group than in the Class II + III group. Notably, the expression level of this gene in the Class II + III obese group was even lower (although not significant) than in the non-obese group. There were no significant differences between the expression level of *SFRP1* in all studied groups (Fig. 2c).

### The inflammatory markers

**Fig. 3:**
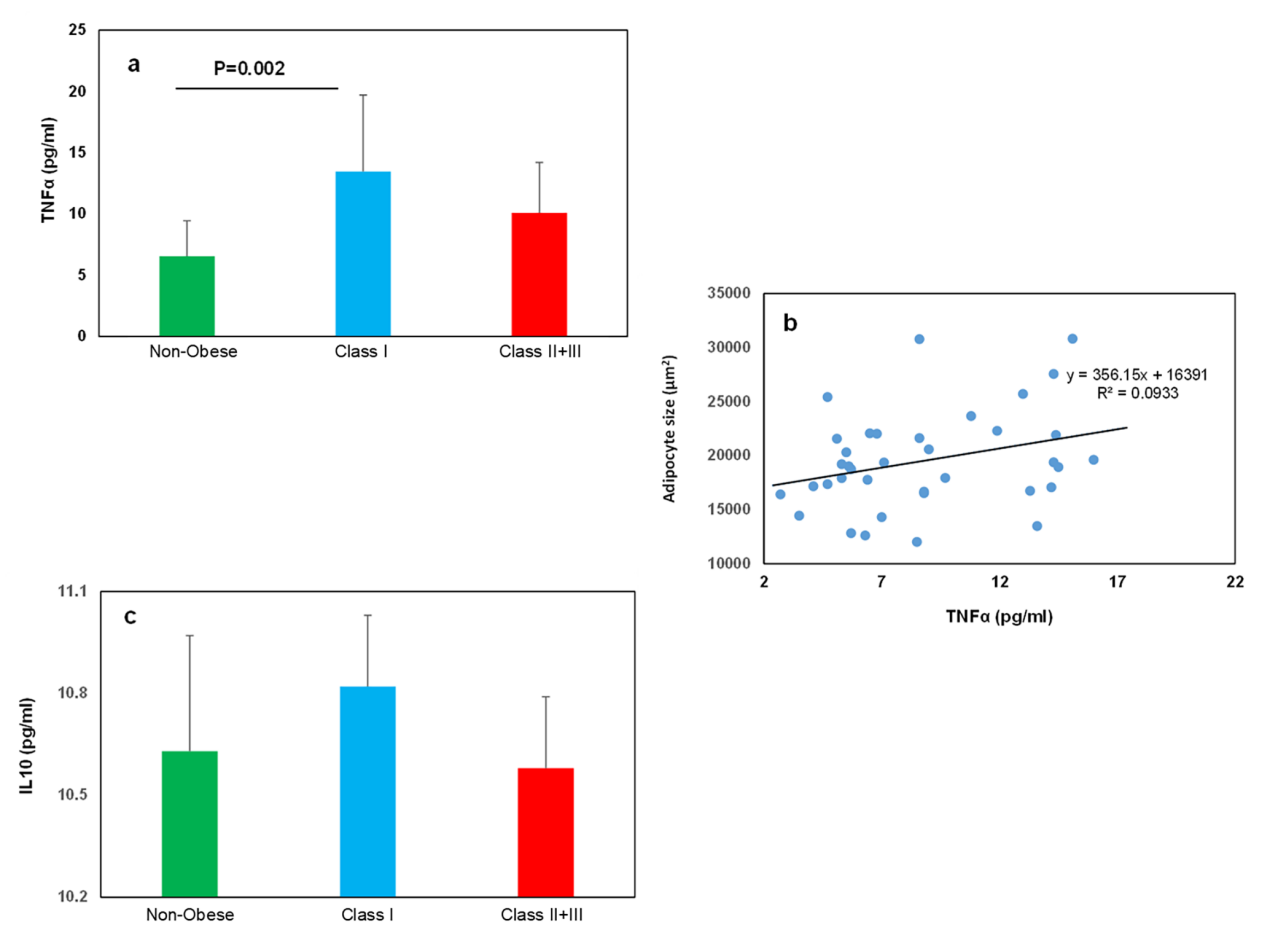
**The serum level of inflammatory markers.** (**a** and **c**) The serum level of TNFα and IL10, respectively, in the studied groups. (**b**) Correlation between TNFα and adipocytes size. All data are shown as mean ± SEM. The level of significance is shown in the histograms

Figure 3a showed that the serum TNFα was higher in the Class I than in the non-obese individuals (0.002). However, there were no significant differences in IL10 levels between groups (Fig. 3c). Figure 3b indicates the association between serum TNFα and adipocyte size (R = 0.01 p = 0.4).

### The expression levels of the sXBP1 gene in scWAT

**Fig. 4:**
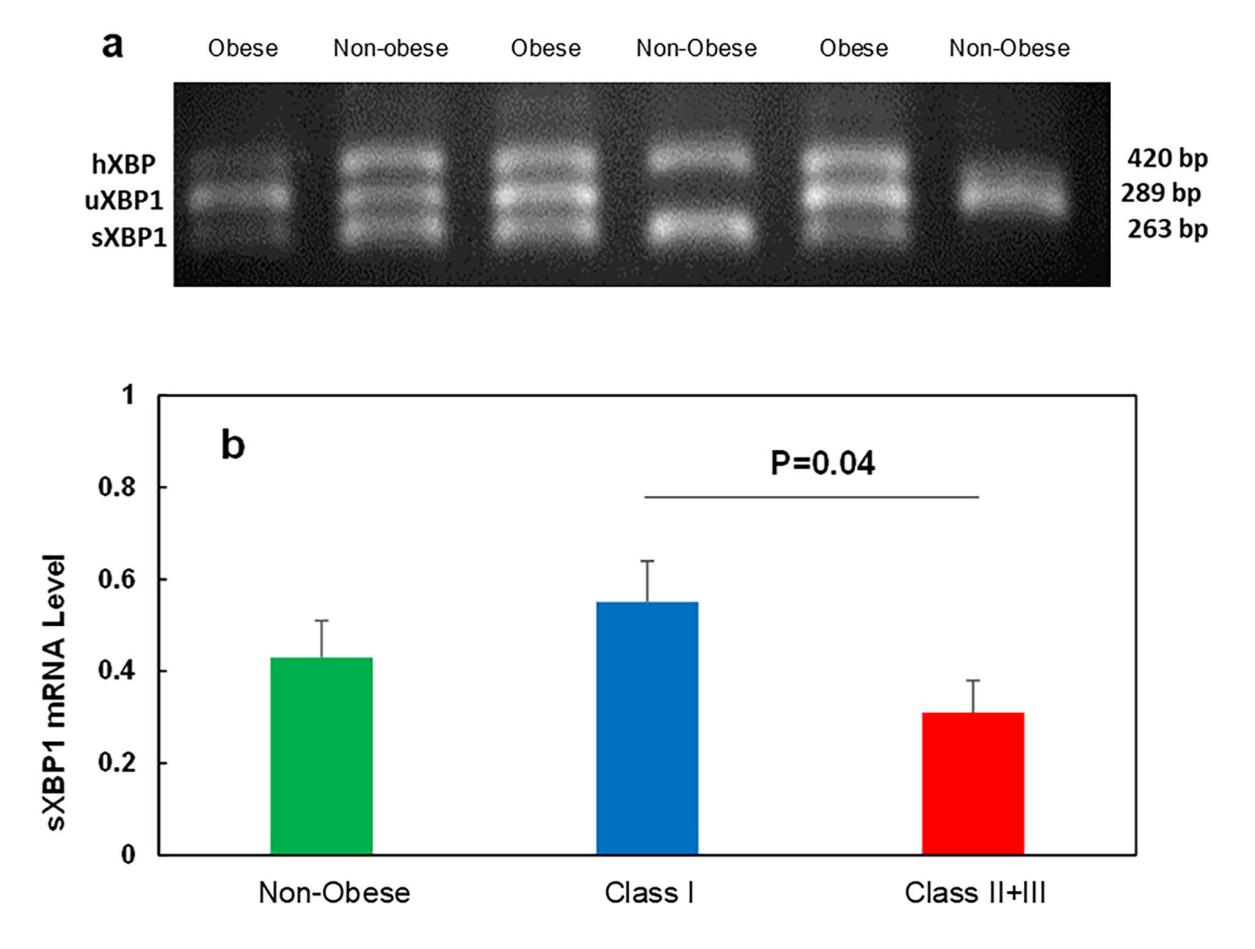
**The level of sXBP1 in scWAT of different groups.** (**a**) Representative image of XBP1 mRNA isoforms. (**b**) mRNA expression of *sXBP1* of non-obese, Class I, and Class II + III Obese groups measured by RT- PCR. All data are shown as mean ± SEM. The level of significance is shown in the histograms

The sXBP-1 levels were evaluated as the main component of the UPR pathway following ER stress. We observed various degrees of sXBP1 expression in the studied groups (Fig. 4a and b). Although there were no significant differences between the sXBP1 expression in the non-obese individuals with obese groups, its expression was significantly (p = 0.04) higher in the adipose tissue of mild obese individuals (Class I) compared to the severely obese individuals (Class II + III).

### VEGFA and CD31 levels in scWAT

**Fig. 5:**
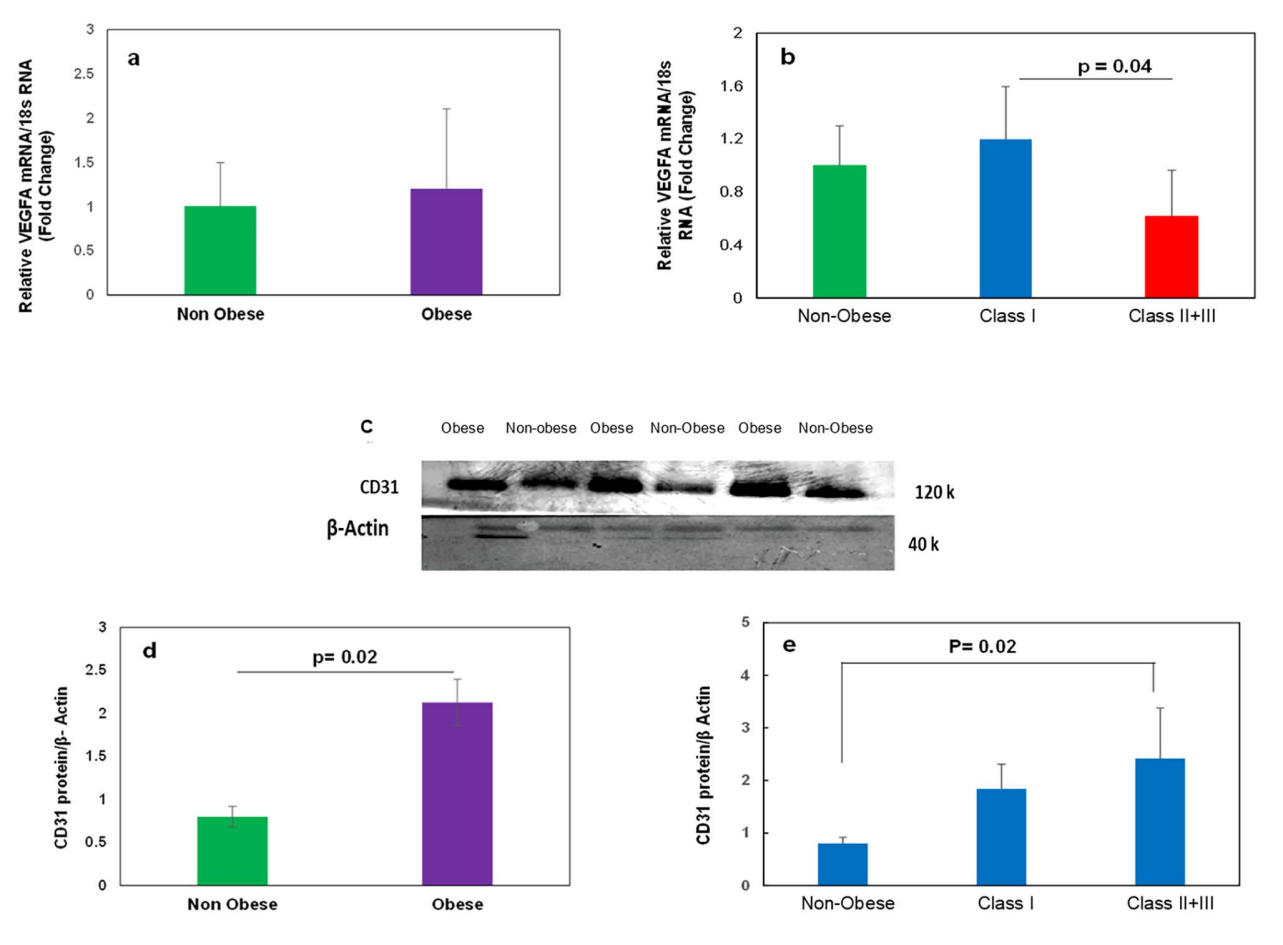
**The level of VEGF mRNA and CD31 protein expression in scWAT.** (**a**) Relative mRNA expression of VEGF in scWAT of non-obese and Obese groups. (**b**) Relative mRNA expression of VEGF in scWAT of non-obese, Class I and Class II + III Obese individuals as measured by quantitative PCR and normalized to 18 s RNA. (**c**) Representative Western blot image of CD31 protein. (**d**) The quantitative analysis of CD31 protein to β-actin ratios in scWAT from non-obese and Obese groups. (**e**) The quantitative analysis of CD31 protein to β-actin ratios in scWAT from non-obese, Class I, and Class II + III Obese groups. All data are shown as mean ± SEM. The level of significance is shown in the histograms

We measured the expression of the *VEGFA* gene, an angiogenic factor, in adipose tissue samples of all individuals. Figure 5a shows a trend of increase in the expression of *VEGFA* in the scWAT of the obese groups compared to the non-obese group. However, Fig. 5b shows a significant difference between Class I obese and Class II + III obese individuals.

Figure 5c shows Western blot spots of CD31, a specific marker of endothelial cells and angiogenesis in adipose tissue. Figure 5d indicates a significantly (p = 0.02) higher level of CD31 protein expression in the obese than in non-obese individuals. However, when we compared the CD31 level between groups, Fig. 5e, a significant (p = 0.02) difference was observed between the non-obese and Class II + III obese group.

### Association between sXBP1, WNT 10B, and VEGFA gene expression

The correlation between sXBP1, WNT 10B, and VEGFA gene expression in the scWAT of the individuals in different classes was analyzed. Significant positive correlations existed between the expression levels of *WNT10*B, *sXBP1*, and *VEGFA*, considering all obesity groups (r = 0.7, p = 0.0001 and r = 0.3, p = 0.01, respectively) (Table 3).

**Table 3:** The correlation between WNT10B, sXBP1 and VEGF gene expression

	VEGF mRNA		sXBP1 mRNA	
	R	P	R	P
WNT10B	0.7	0.0001	0.6	0.0001
sXBP1	0.3	0.001		

## Discussion

This study evaluated the metabolic, ER stress, and angiogenic parameters concerning the hypertrophy/hyperplasia of scWAT in individuals with different BMI levels. Our results indicated an aberrant metabolic profile in obese individuals relative to the non-obese group significantly associated with the size of scWAT adipocytes. Adipocyte sizes were significantly larger in Class I obese individuals with higher serum TNFα, insulin levels, and HOMA-IR values. Furthermore, all the studied cases showed positive correlations between the scWAT adipocyte size, serum TNFα, insulin levels, and HOMA index. Therefore, the adipocyte size is a more critical indicator in the glycemic parameters of obesity than the extent of adiposity. In agreement with these results, the positive correlation between adipocyte size in the visceral and subcutaneous adipose tissue with TG and insulin levels and a negative correlation with HDL and insulin sensitivity has been reported previously [20]. It has also been reported that adipocyte size could be an insulin resistance index independent of BMI [21].

It has been found that people’s capacity in adipogenesis and harmless storage of the mounted lipids is different based on genetic, racial, and gender variables [22]. In addition to other parameters such as dietary intake, lifestyle, and physiological and pathological states, proper adipogenesis is the primary determinant of the healthy expansion of adipose tissue. In situations where the lipid load exceeds the capacity of adipose tissue expansion and safe storage of lipids, excessive fat causes hypertrophy of the scWAT adipocytes and ectopic damaging fat accumulation. Moreover, these conditions are associated with inflammation and aberrant metabolic outcomes [23, 24].

Based on our findings, the *PPARγ2* expression as a primary adipogenesis regulator in scWAT was significantly higher in the Class II + III obese group, following their higher BMI values. In addition, the level of *WNT10B* expression, as a negative regulator of adipose tissue expansion, was lower in this group than in Class I obese group. In contrast, individuals with Class I obesity with lower levels of *PPARγ2* and higher levels of *WNT10B* would have a lower capacity for tissue expansion in response to lipogenesis and excessive loads of lipids. In this situation, excess fat causes the hypertrophy of the current adipocytes. Consistent with our results, other studies reported the role of the proadipogenic *PPARγ2* and the antiadipogenic of *WNT10B*. A negative relationship between preadipocyte differentiation and adipocyte size was found in human abdominal obesity. In comparison, the PPARγ expression was high in preadipocytes [25]. The lower expression of PPARγ, as a gene encoding marker of adipose cell differentiation, has also been reported in scWAT of obese individuals with insulin resistance [26]. The decreased PPARγ, as the adipogenic factor, is associated with the decreased mediators of insulin signaling in adipose tissue [27]. Along with PPARγ, the role of WNT10 has also been considered in adipogenesis. The transgenic induction of Wnt10b in animals receiving a high-fat diet inhibited the development of adipose tissues and weight gain [12]. In agreement with these findings, hypoxic induction of WNT10B expression in adipogenic cells inhibited adipogenesis of human mesenchymal stem cells [28].

*SFRP1*, as an adipogenic factor, was another gene we tested for its expression. The adipogenic effect of *SFRP1* is apparently mediated by Wnt pathway inhibition in adipose tissue. In a study with a broad range of BMI participants, a lower level of *SFRP1* expression and a negative linear relationship with BMI has been reported in obese individuals [29]. However, our results showed no significant differences in the expression of this gene in different groups, possibly due to the limited number of individuals.

In Class I obesity cases, the increased insulin level, HOMA index, and sXBP1 levels were consistent with the hypertrophy of adipocytes and their unfavorable metabolic status. However, the ER stress marker and insulin resistance were not elevated in Class II + III obese individuals, despite the higher adiposity and BMI values. In line with these results, the decreased expression of adipogenic genes in obese individuals with type 2 diabetes compared to non-diabetic obese individuals has been reported [30].

It has been shown that adipose tissue expansion during adipogenesis requires adequate development of the blood vessels and angiogenesis. In the current study, we found higher levels of CD31 (a marker of microvessel density) in Class II + III obese groups with higher scWAT hyperplasia than in Class I obese cases with hypertrophic adipocytes. This result confirms the previous reports regarding the association of adipose tissue expansion with angiogenesis. Proportional vascular development with adipogenesis ensures adequate infusion and prevents the consequences of inadequate angiogenesis, including hypoxia, inflammation, and metabolic disorders [31, 32]. This requirement was satisfied with the Class II + III obesity group. However, in Class I, the non-proportional fat accumulation regarding the lower adipogenic capability and unmatched blood vessel density (lower CD31 levels) could result in hypoxia, ER stress, and metabolic impairment. The findings were confirmed by the induced VEGFA, spliced XBP1, and insulin resistance in Class I obese individuals. As the central mediator of angiogenesis, VEGFA is induced in suboptimal perfusion and hypoxia conditions that give rise to endothelial cell proliferation and vessel sprouting [33]. The VEGFA expression was higher in Class I obese group, suggesting insufficient blood flow into the adipose tissue with hypertrophic cells. The induced VEGFA levels correlated significantly with the elevated sXBP1 levels. This result is in line with other studies that show the effect of sXBP1 on VEGFA gene expression [8]. Some previous studies focused on the importance of angiogenesis in the healthy development of adipose tissue and the associated metabolic status [34, 35]. It has been shown that targeting angiogenesis by modulating VEGFA levels could influence adipose tissue expansion and metabolic variables such as insulin resistance [36, 37].

In addition, our previous study highlighted the critical role of WNT10B in adipose tissue angiogenesis. As a positive regulator, we showed that WNT10B promotes the formation of new vessels through the upregulation of VEGFA expression [38]. In the present study, we observed a significant increase in the expression of these two genes in Class I obesity, which might be induced by ER stress and XBP1 splicing. These changes were accompanied by a metabolic disorder, insulin resistance, and inflammation in those with hypertrophic scWAT adipocytes and limited adipose tissue expansion.

The present study’s limitations include the participants were healthy individuals under elective surgery. In addition, these cases were not evaluated for nutritional status, the dietary intake of carbohydrates and fat calories, or lifestyle parameters such as physical activity. Therefore, we have also suggested performing a similar study with a larger sample size of participants in both males and females with different BMI levels.

## Data Availability

All the data will be available on request.
